# Exploring prevalence and factors associated with postpartum depression among Ukrainian women

**DOI:** 10.18332/ejm/188800

**Published:** 2024-07-02

**Authors:** Nataliia Gusak, Sally Kendall, Olena Nizalova

**Affiliations:** 1 School of Social Work, National University of Kyiv Mohyla Academy, Kyiv, Ukraine; 2 Centre for Health Services Studies, University of Kent, Canterbury, United Kingdom

**Keywords:** postpartum depression, LMIC, maternal mental health

## Abstract

**INTRODUCTION:**

Postpartum depression negatively impacts maternal mental health and child development. The high prevalence of postpartum depression (PPD) in low and lower middle-income countries raises questions about its predictors. This study examines the association between PPD and breastfeeding experience, child death, unresolved pregnancy, forced displacement, COVID-19 pandemic lockdown, marital, and financial status among Ukrainian women.

**METHODS:**

This online study recruited 1634 Ukrainian mothers of children aged 0–5 years through non-governmental organizations providing services to them. The Edinburgh Postnatal Depression Scale (EPDS), with a cut-off of ≥13, was used to assess depressive symptoms in the postpartum period. Independent t-tests, chi-squared tests, one-way ANOVA, non-parametric correlations, and logistic regression tests were used to analyze the data.

**RESULTS:**

The prevalence of depressive symptoms was 39.0% (n=1631). There was a positive association between EPDS scores and breastfeeding difficulties, pandemic lockdown, and financial difficulties. We did not find an association between PPD symptoms and unresolved pregnancy, death of a child, being affected by COVID-19, and forced displacement. We found that EPDS scores for women who did not experience forced displacement (n=1528) were significantly higher compared to displaced mothers (n=74).

**CONCLUSIONS:**

The present study of Ukrainian women shows that women experienced depressive symptoms influenced by various factors including breastfeeding difficulties, pandemic lockdown, and financial difficulties. There is a need for additional research into such factors as unresolved pregnancy, the death of a child, being affected by COVID-19, and forced displacement.

## INTRODUCTION

Pregnancy and the postpartum period are associated with women’s physical and emotional disturbance, and in one in five cases, leads to the development of postpartum depression (PPD)^1^. The global prevalence of PPD is about 17.7%, but this can vary by nation^2^. Cultural and economic differences play a significant role in PPD^3^, with a prevalence of 10% to 15% in Western countries and 18.6% in low- and middle-income countries^4^. Ethnic inequalities also matter^5^. In addition, non-indigenous, indigenous, and immigrant women within high-income countries have significantly different PPD scores^6^. Despite the high prevalence of PPD worldwide, many cases are not diagnosed and remain untreated, resulting in negative consequences for mother and child^7^. Maternal mental health is critical for developing a child’s healthy brain and physical, social, and emotional well-being in the first 1001 days, from conception to two years.

There are several predictors of postpartum depression described in the literature. The most significant contributors to PPD are antenatal depression and anxiety, previous psychiatric illness, poor marital relationship, being young, being unmarried, negative attitude towards pregnancy, lack of social support, and stressful life events^8,9^. These events can include natural disasters, growing up with divorced parents, losing a loved one unexpectedly, and experiencing emotional or physical abuse^10^. Studies also suggest that women with a prior pregnancy loss were 35% more likely to require post-partum psychiatric treatment^11^. Furthermore, bereaved women had nearly 4-fold higher odds of having a positive screen for depression^12^. The prevalence of PPD in women was relatively high during COVID-19^13^, and the pandemic highlighted existing barriers to the treatment of peripartum mood disorders^14^. Additionally, the prevalence of perinatal depressive disorders was 24.2% among all women who are migrants and 32.5% among displaced people^15^.

In Ukraine, one in three people experienced at least one mental health problem in their lifetime; depression was prevalent in 6% of the general population; apparent gender differences are also seen – anxiety disorders and depression are more prevalent among women compared to men^16^. Ukraine has a highly centralized single-payer healthcare system, where healthcare is free for everyone^17^. In this system, mental health treatment was available in big psychiatric institutions with limited access to services at the primary level in the community. In 2017, Ukraine started reforming the health system by changing the government’s financial guarantees of public medical services^18^. The changes made in the mental health system led to significant budget cuts for most state psychiatric hospitals. This resulted in mental health services being provided through primary healthcare and community services. However, this created challenges for the primary healthcare system as there was limited training and managing responsibilities between professionals^19^. Although evidence-based interventions for mental health treatment are slowly being integrated into service provision, mental healthcare for women during their perinatal journey is still a concern. Several professionals such as family doctors, outpatient and inpatient gynecologists, and breastfeeding consultants provide services during this period. However, only a few of them have received training in perinatal mental health and can respond to women’s mental health needs. Therefore, many women seek help from non-governmental organizations in their local communities or online, relying on peer-to-peer support.

There is limited epidemiological data on maternal mental health in Ukraine. However, some information is available about displaced mothers. A significant fraction of the Ukrainian population has been displaced by force^20^, with relocation of civilian women and children^21-23^. The consequences of migration can be particularly damaging for mothers and babies. Studies have shown that the trauma of forced displacement can have long-term adverse effects on mental health, leading to an increased risk of depression and post-traumatic stress disorder^24^. Displaced pregnant women in Ukraine had a 34.8% frequency of post-traumatic stress disorder^25^, 3.3 times higher reactive anxiety, and 2.6 times higher personal anxiety^26^. There is also evidence indicating that forcefully displaced populations have an increased risk of reactive and personal anxiety, depressive manifestations, autonomic dysfunction, insomnia, and premature termination of pregnancy^27^. The ongoing research aims to investigate the impact of forced displacement on perinatal mental health, including anxiety, post-traumatic stress, depression, and birth trauma symptoms^24^. However, the other factors associated with PPD among Ukrainian mothers are not well understood in the literature. The purpose of this study is to understand the association between PPD and various potentially stressful life events, such as breastfeeding difficulties, child death, unresolved pregnancy, forced displacement, COVID-19 pandemic lockdown, and marital and financial status, among Ukrainian women.

## METHODS

### Design and study setting

The data for the current analysis was obtained from a more extensive cross-sectional study conducted by the authors on Perinatal Mental Health in Ukraine (PMHUa-2020). Before this study, researchers conducted a 2-day workshop on maternal and infant health in June 2019 in Kyiv to develop strong partnerships with local service providers and experts. This study was conducted among Ukrainian women on their perinatal journey receiving services from non-governmental organizations who agreed to support the study. Data were collected during February–August 2020 through an online survey in Qualtrics designed by the research team.

### Sampling and study populations

This mixed-methods study was conducted among mothers of children aged 0–5 years from most oblasts of Ukraine. The number of participants required for this analysis was calculated using G-power software. The sample size calculation suggested that 128 participants would provide 80% power in an independent sample t-test (alpha=0.05) to detect a significant difference in PPD between women who experienced forced displacement and those who have not; 143 participants – for chi-squared test to find statistical significance in the relationship between financial difficulties and COVID-19 lock down impact; 200 participants – for a one-way ANOVA to test differences in women’s PPD scores based on marital status; 128 participants – for non-parametric correlation analysis to examine the association between PPD and household income per month; and 568 participants – for logistic regression analysis. With 1634 responses, there was sufficient power to find statistical significance using the suggested analysis.

### Inclusion and exclusion criteria

The inclusion criteria were women who self-identified as Ukrainians, could read and write Ukrainian, were aged ≥18 years, had children aged 0–5 years, and were willing to participate in the study. We focused on the first five years because this is a critical time for child development, especially in developing countries^28^. The study excluded women who could not read and write Ukrainian, were aged >18 years, and had children aged >5 years.

### Measurement

Women received a link to an online questionnaire in Qualtrics, which included sociodemographics, women’s recent experiences related to mental health issues, and services or interventions. The questionnaire was translated into Ukrainian and reviewed by the mental health and maternal health experts in Ukraine to ensure it covered the range of topics to achieve the study objectives, that questions were clear, culturally relevant, and that the wording was easily comprehended. The questionnaire included items related to breastfeeding, child death, unresolved pregnancy, forced displacement, COVID-19, pandemic lockdown, marital status, financial difficulties, and household income. In addition, the questionnaire incorporated the Edinburgh Postnatal Depression Scale (EPDS), a self-reported scale composed of 10 items of common depressive symptoms that uses a Likert-type format for the responses^29^. Answers ranged from 0 to 3 (questions 1, 2, and 4 had reverse order), and possible scores ranged from 0 to 30. A cut-off point of ≤10 represents a minor risk to PPD; a cut-off of ≥13 represents medium to high risk. During the study, we inquired about the symptoms that the participants experienced, not immediately after childbirth. The EDPS questionnaire was translated into Ukrainian and validated by The Ukrainian Institute of Cognitive Behavior Therapy in 2021. For this study, we used a score of 13 as the cutoff for identifying the risk of PPD.

### Procedures

Several non-governmental organizations provide services to women in the perinatal journey, including ‘Molochni Riky’, Life Support and Maternity Club ‘Lada’, ‘Terytoria Mam’, ‘Ranni Ptashky’, International Leadership and Development Centre, ‘Kolo Simji’, Center for crisis pregnancy House of Hope, 280 Days, Angels Care, ‘Poslanets Myru’, More for Ma, X-Mothers Club, and ‘Dbaylyvo do Sebe’ Project, which agreed to support the study. These NGOs sent mothers information about the research through emails, groups on social media, and chats they supported. Those who agreed to participate and met the inclusion criteria signed an online informed consent form and completed an online survey in Qualtrics, which took around 30 minutes. All women participating in the survey were informed about available service providers where they can get support for their mental health. Overall, 1634 participated in the study.

### Analysis

We conducted our statistical analysis using the SPSS 27.0.1.0 for the IOs package. We calculated the cut-off point for EPDS ≥13. There were six extreme cases with scores ≥28. However, these data might represent severe cases and were not excluded from the analysis. We used the one-sample t-test to compare the prevalence of postpartum depression as measured by the EDPS between Ukrainian women and women in low- and lower middle-income countries. Because we did not have the nationally representative data about the prevalence of PPD, we used previous research data from the Wang et al.^4^ meta-analysis about the minimum average prevalence of PPD for women in LMICs (18.6%). In order to examine the difference in PPD scores between women who experienced forced displacement and those who have not, we performed an independent sample t-test. We used chi-squared analysis to examine the relationship between financial difficulties and COVID-19 lockdown impact. We conducted one-way ANOVA to test differences in women’s PPD scores based on marital status. Non-parametric correlation analysis was used to examine the association between PPD and household income per month. We performed a multivariate logistic regression analysis to examine which potential stressful life events were predictive of postpartum depression scores. We conducted power analysis to indicate sample size and found that there was sufficient power to find statistical significance by the above-mentioned tests.

## RESULTS

Table 1 presents the characteristics of study participants. This analysis used the final sample of 1634 women who participated in the online survey during their perinatal period.

**Table 1:** Selected characteristics of the study participants, Ukraine, 2020 (N=1631)*

*Characteristics*	*n (%)*
**Level of postpartum depression score,** mean (SD)	11.41 (6.09)
**Existing postpartum depression score**	
≤12	994 (60.8)
≥13	637 (39.0)
**Breastfeeding difficulties**	
No trouble	297 (20.8)
A little trouble, resolved without any help	541 (37.8)
A little trouble, resolved with help	406 (28.4)
So much trouble	186 (13.0)
**Child death**	
Yes	16 (2.3)
No	692 (97.7)
**Unresolved pregnancy**	
Yes	320 (45.2)
No	388 (54.8)
**Forced displacement**	
Yes	74 (4.6)
No	1,534 (95.4)
**COVID-19**	
Yes	51 (3.1)
No	1576 (96.9)
**Pandemic lockdown** (anxious/afraid about it)	
Constantly	72 (4.4)
Most days	196 (12.1)
Sometimes	884 (54.4)
Hardly ever	226 (13.9)
Not at all	247 (15.2)
**Marital status**	
Single	31 (1.9)
Married	1529 (93.7)
Widowed/divorced/separated	71 (4.4)
**Financial difficulties**	
Not enough money for food even	27 (1.7)
Enough money just for food	291 (18.0)
Enough money for food and clothes, cannot buy expensive things	532 (32.9)
Can afford to buy some expensive things or save money	580 (35.8)
Can make significant savings	189 (11.7)
**Household income** (UAH)	
≤10000	575 (37.0)
10001–30000	583 (44.0)
>30000	290 (19.0)

* N=1631 postpartum depression, marital status data. N=1430 for breastfeeding data. N=708 for child death data and unresolved pregnancy data. N=1608 for forced displacement data. N=1627 for COVID-19 affected data. N=1625 for pandemic lockdown data. N=1619 for financial difficulties data. N=1553 for household income data. UAH: 1000 Ukrainian Hryvnia about US$370, at the time of the study.

Almost all survey participants (93.7%) were married, only 4.4% were divorced/separated/widowed, and 1.9% were single. Assessing their financial difficulties, only 11.7% respondents were able to make significant savings; 35.8% to buy some expensive things or save money; 32.9% had enough money for food, clothes and could save a little; 18% of women have enough money just for food, and 1.7% did not have enough money even for food. About 4.6% of women recognized themselves as internally forcefully displaced. About 13% of women reported severe troubles with breastfeeding; 28.4% had a little trouble and resolved it with help. More than one-third of respondents (37.8%) had a little trouble and resolved it without any help. About 20.8% did not have trouble with breastfeeding at all. Only 2.3% of respondents experience the death of a child. A little bit less than half of respondents (45.2%) had a pregnancy that did not end in a live birth because of a miscarriage, an abortion, or the child was stillborn. While 54.8% did not have such experience with unresolved pregnancy. Only 3.1% of respondents recognize themselves and their family members as those who were affected by COVID-19. The pandemic lockdown made 4.4% of study participants constantly feel anxious/afraid about the COVID-19 pandemic, and 12.1% felt anxious/afraid most days but could find ways to distract themselves. More than half (54.4%) felt anxious/afraid sometimes. About 13.9% of the sample described themselves as hardly ever anxious/afraid, and 15.2% indicated they were not anxious/afraid.

We found that 39.0% of respondents scored ≥13 on the EPDS, which indicates postpartum depression symptoms. Valid EPDS scores ranged from 0 to 30 with a mean of 11.41 (SD=6.09). The data for PPD scores is approximately normally distributed with skewness of 0.407 and kurtosis of -0.319.

In the independent sample t-test, we found that mean EPDS scores for 1528 women who did not experience forced displacement (mean=11.44, SD=5.99) were significantly higher than mean EPDS scores for the 74 women who experienced forced displacement (mean=9.31, SD=5.69). The results indicate that women who experienced forced displacement have a PPD score of 2.20 less than women who have not experienced forced displacement [t(1606)=3.05, p<0.01] with a moderately small size effect (Cohen’s d=0.363).

In the chi-squared test, we found that there was a significant relationship between financial difficulties and pandemic lockdown (χ^2^=66.34, p<0.001), with a weak effect (Cramer’s V=0.101). Those who can afford to buy some expensive things or save money hardly ever feel anxious/afraid about pandemic lockdown (41.7%, n=93). Women who had enough money for food and clothes but could not buy expensive things also felt anxious/afraid about pandemic lockdown most days (39.5%, n=77). The number of those who could make savings and did not feel anxious/afraid about the pandemic lockdown at all was small (n=32). Also, a small number of women did not have enough money even for food and felt constantly anxious/afraid about the pandemic lockdown (n=8). Even though this is the smallest group of people (those who selected ‘we do not have enough money for food even’), almost one-third were always afraid, while for all the richer groups, less than 7% reported being always afraid.

Based on one-way between-subjects ANOVA, Welch’s test indicated a significant difference in women’s PPD scores based on their marital status [F (2;5836)=3.23, p<0.05], with a small size effect (η^2^=0.005). Divorced/separated/widowed women had the highest level of postpartum depression (mean=13.25, SD=7.29). A Games-Howell *post hoc* test did not indicate significant differences in depression scores between these groups.

In non-parametric correlation, we found a small positive correlation between women’s PPD scores and household income per month (Spearman’s rho=0.112, p<0.001). The relationship was positive, indicating the household income per month increased, and so did PPD scores. Women who had higher household income were more likely to experience postpartum depressive symptoms (Table 2).

**Table 2:** Spearman’s rho correlations between study independent variables and PPD using EPDS-13, Ukraine, 2020 (N=1631)

*Variables*	*1*	*2*	*3*	*4*	*5*	*6*	*7*	*8*	*9*
1. Breastfeeding	-								
2. Child death	0.000	-							
3. Unresolved pregnancy	-0.082*	0.005	-						
4. Forced displacement	0.024	-0.027	0.058	-					
5. COVID-19	0.004	0.034	0.043	-0.005	-				
6. Pandemic lockdown	-0.088**	-0.012	-0.036	0.000	0.068**	-			
7. Financial difficulties	-0.076**	0.042	0.017	0.005	-0.009	0.096*	-		
8. Household income	-0.018	0.037	-0.017	-0.033	-0.025	0.042	0.609**	-	
9. PPD	-0.209*	0.023	-0.023	-0.073**	0.003	0.243**	0.173*	0.112**	-

* p<0.05.

** p<0.01.

PPD: postpartum depression. EPDS: Edinburgh Post-natal Depression Scale with a score ≥13 as the cutoff.

The multivariate logistic regression model was statistically significant [χ^2^(15)=59.67, p<0.01]. The model explained 12.5% (Nagelkerke R^2^) of the variance in postpartum depression and correctly classified 61.0% of cases. The predictors are presented in Table 3. Significant breastfeeding characteristics included having so much trouble that women were not able to breastfeed, having a little trouble that was resolved with help, and having a little trouble that was resolved without any help. Women having so much trouble that they were not able to breastfeed were 3.5 times more likely to have postpartum depression than women who had no trouble at all. Women having a little trouble that was resolved with help were 2.0 times more likely to have postpartum depression. Women having a little trouble that was resolved without any help were 2.1 times more likely to have postpartum depression.

**Table 3:** Multivariate logistic regression predicting whether or not postpartum depression occurs, Ukraine, 2020 (N=1631)

*Predictor*	*B*	*SE*	*OR*	*95% CI*
**Breastfeeding difficulties** (ref. no trouble at all)				
So much trouble, not able to breastfeed	1253	0.317	3.502***	1.882–6.519
A little trouble, resolved with help	0.714	0.262	2.042**	1.223–3.410
A little trouble, resolved without any help	0.735	0.233	2.085**	1.320–3.293
**Pandemic lockdown** (ref. feeling anxious/afraid constantly)				
Not at all	-1.433	0.473	0.239**	0.095–0.603
Hardly ever	-1.565	0.481	0.209***	0.081–0.537
Sometimes	-1.637	0.430	0.195***	0.084–0.452
Most days	-0.935	0.476	0.392*	0.154–0.997
**Financial difficulties** (ref. not have enough money for food even)				
Can make significant savings	-1.548	0.769	0.213*	0.047–0.959
Can afford to buy some expensive things or save money	-1.238	0.724	0.290	0.070–1.199
Enough money for food and clothes, cannot buy expensive things	-0.997	0.726	0.369	0.089–1.532
Enough money just for food	-0.861	0.731	0.423	0.101–1.770
**Unresolved pregnancy** (ref. yes)	0.060	0.177	1.061	0.751–1.500
**Death of a child** (ref. yes)	-0.553	0.675	0.575	0.153–2.161
**COVID-19 affection** (ref. yes)	-0.253	0.528	0.777	0.276–2.187
**Forced displacement** (ref. yes)	-0.445	0.524	0.641	0.229–1.790

* p<0.05.

** p<0.01.

*** p<0.001.

Significant pandemic characteristics are feeling anxious/afraid not at all, hardly ever feeling anxious/afraid, feeling anxious/afraid sometimes, and feeling anxious/afraid most of the days. Women who were afraid all the time were five times more likely to experience PPD compared to women who did not feel afraid at all, and 2.5 times more compared to women who felt anxious/afraid most of the day. Significant financial difficulties were described as being unable to buy expensive things or save money. Women who did not have enough money for food were five times more likely to experience PPD compared to women who could afford to buy some expensive things or save money. The unresolved pregnancy, death of a child, COVID-19 affection, and forced displacement were not associated with postpartum depression.

## DISCUSSION

In this study of pregnant Ukrainian women and mothers who gave birth during 2016–2020, we found: 1) that the prevalence of PPD in this sample was higher compared to the average PPD scores in LMICs; 2) a positive association between PPD scores and such potential stressful life events as breastfeeding difficulties, pandemic lockdown, and financial difficulties; and 3) no association between PPD scores and unresolved pregnancy, death of a child, being affected by COVID-19, and forced displacement.

We found that the prevalence of postpartum depression among women participating in the survey was higher compared to the prevalence of postpartum depression among women in LMICs, where it is considered to be 18.6%^4^. The variability in PPD prevalence might be caused by cross-cultural variables, differences in perception of mental health, and its stigma^30^. According to Quirke et al.^31^, there is a high lack of knowledge and understanding about most types of mental illnesses among adults in Ukraine and a high degree of social distance. There are beliefs that seeking help may be a sign of weakness. It can be explained that the image of mental health has been associated with political abuses in psychiatry and stigmatizing attitudes, and discriminating practices against people with any form of disability^32^. However, women in our study were invited to participate in it by non-governmental organizations providing support for new mothers. They might have been more aware of maternal mental health compared to the general population.

The prevalence of PPD symptoms (39%) among females within our sample is in the range of prevalence of PPD (from 0% to 60%) reported in the literature on PPD scores in non-Western countries^30^. While the studies might use different reporting styles^33^, the Edinburgh Postnatal Depression Scale (EPDS) has been widely used for detecting perinatal depression and research for over 30 years and has been translated into 60 languages, including Ukrainian. While it is the most popular screening tool, there is a discussion that it is misused, and its authors recommend future studies to evaluate its use and validity in naturalistic community populations and to determine the psychometric properties and practical usefulness of the EPDS when completed online^29^. Despite using EPDS in our online study, women who participated in it were at different stages of their perinatal journey – from being pregnant to being a mother of children aged 0–5 years – and talked about their symptoms during the study. We collected data at the pandemic’s beginning, which may have affected the study results. Chen et al.^34^ highlighted that the pooled prevalence of PPD during the COVID-19 pandemic was 34%, which is much higher than those from previous research during the non-pandemic period.

Our second finding contributes to the literature on predictors of PPD symptoms. Breastfeeding difficulties, pandemic lockdown, financial difficulties, divorce, separation, or death of a spouse were all found to be risk factors for PPD, and this is consistent with literature review and meta-analysis of the prevalence and risk factors associated with PPD during the COVID-19 pandemic^34^.

The association between breastfeeding difficulties and PPD symptoms is in line with previous studies before and during the pandemic^34^. However, while the association between breastfeeding difficulties and PPD has been found previously, numerous studies on the topic of breastfeeding and postpartum depression have come to contrasting conclusions as to why this association exists^35^. Some studies indicate the importance of educating nurses, family doctors, and family members to reduce the risk of postpartum depression in line with the UNICEF standards^36^. Other studies explain that the stress associated with medical and societal pressures to breastfeed could impact mothers’ mental health.

We found an association between PPD scores and the pandemic lockdown that was in line with the Chen et al.^26^ literature review and meta-analysis. Women reported that they were anxious/afraid during the pandemic lockdown, which can explain their financial situation. In this study, we found a very strong association between poverty and the mental health impacts of lockdowns. Every fifth respondent needed to meet the subsistence level for a mother and one child. To compare, the subsistence level in 2020 was UAH 2481 (US$91) for one adult and UAH 2100 (US$78) for a child under six years old. However, more research is needed as the number of household members and income per person may vary.

An unresolved pregnancy, death of a child, being affected by COVID-19, and forced displacement were not associated with postpartum depression in our study. Also, there was nothing about this in the Chen et al.^34^ review. We hypothesize that internally forcefully displaced women might be more resilient and resistant to stress, and more research is needed to explore this possibility. Additional study is needed to examine the association between PPD and COVID-19 affections. Because the data were collected at the beginning of the pandemic and was affected by COVID-19, it might be understood differently since the cases increased across Ukraine during the pandemic and inadequately sampled during the internal forced displacement.

### Limitations

This study has some limitations. We utilized data from a cross-sectional, non-representative online survey. Our study consisted of women from different regions of Ukraine, with most subjects being highly educated females from large cities. These demographic characteristics are comparable to the general population, where almost 92% of females are educated, and about 70% are urban residents. However, it is essential to note that the current study may only represent some Ukrainian women as it excludes those who do not have access to the internet or have minimal online activity. As of 2020, the internet penetration rate in Ukraine was 79%, meaning that at least 21% of Ukrainian women could not participate in the survey.

Furthermore, a few cases were internally displaced, which may affect the study’s representativeness. For this study, we rely on self-reported data using the Edinburgh Postnatal Depression Scale (EPDS), a widely used screening tool for assessing postpartum depression symptoms. The study only includes participants who received services from a non-governmental organization during the perinatal period. It does not account for women who did not receive NGO support during their perinatal journey. This may result in a bias towards women who are more aware of mental health issues compared to the general female population. However, unlike most studies on perinatal mental health, which gather data from clinical settings, this research includes those not represented in clinical records. It is important to note that the data were collected at the beginning of the COVID-19 pandemic and, as such, may not be sufficient to make assumptions about the impact of the pandemic on postpartum depression.

## CONCLUSIONS

This study is the first of its kind to examine the factors that contribute to postpartum depression (PPD) among Ukrainian women. Our findings suggest that Ukrainian mothers experience symptoms of PPD that are associated with potentially stressful life events such as difficulties with breastfeeding, pandemic lockdown, financial struggles, and divorce/separation/death of a spouse. However, we did not find associations between PPD scores and unresolved pregnancy, death of a child, being affected by COVID-19, or forced displacement. It is important to note that multiple stressors may interact and negatively impact maternal mental health. Therefore, further research is needed to understand the effects of various stressors, particularly during forced displacement. This study highlights the importance of understanding the predictors of postpartum depression among Ukrainian mothers, which should be included in the training and continuing professional development of various professionals in Ukraine. This includes family doctors, outpatient and inpatient gynecologists, and breastfeeding consultants who aim to address the mental health needs of women.

## Data Availability

The data used to support the findings of this study are restricted by the the University of Kent, School of Sociology, Social Policy and Social Research Ethics Committee in order to protect women privacy. Data are available from Centre for Health Services Studies, University of Kent, Canterbury, CT2 7NZ, United Kingdom for researchers who meet the criteria for access to confidential data.
